# Navigating regulatory scenarios for accelerating drug repurposing in rare diseases: a multi-stakeholder perspective on the challenges and opportunities

**DOI:** 10.1186/s13023-026-04551-8

**Published:** 2026-08-14

**Authors:** Esther A. M. Bührman, Martina C. Cornel, Sofia Oliveira-Martins, Martin de Kort, Wouter P. C. Boon, Tessel Rigter

**Affiliations:** 1 Section Community Genetics, Department of Human Genetics, Amsterdam UMC, Location Vrije Universiteit, Amsterdam, The Netherlands; 2 Amsterdam Public Health Research Institute, Personalized Medicine, Amsterdam, The Netherlands; 3 Faculty of Pharmacy of the University of Lisbon (FFUL), Lisbon, Portugal; 4 Comprehensive Health Research Centre, University of Évora, Évora, Portugal; 5 European Infrastructure for Translational Medicine (EATRIS ERIC), Amsterdam, The Netherlands; 6 Copernicus Institute of Sustainable Development, Utrecht University, Princetonlaan 8a, Utrecht, 3584 CS The Netherlands

**Keywords:** Drug repurposing, Rare diseases, Signature-based, iPSC, Basket trial, Regulation, Marketing authorisation, Pharmaceuticals, Multi-stakeholder dialogue, SIMPATHIC

## Abstract

**Background:**

The lack of treatments for patients with a rare disease calls for innovative approaches in drug development. A potential solution to accelerate therapy development is to find new therapeutic uses for existing drugs, i.e. drug repurposing. One way of advancing drug repurposing is to focus on similarities in the molecular and clinical manifestations across multiple rare diseases, by studying drug repurposing candidates in induced pluripotent stem cell (iPSC) models and subsequently grouping diseases in basket trials composed of multiple sub-studies. However, the absence of a single route to navigate the regulatory process, in combination with limited regulatory experience in academia poses a challenge for this approach. To provide more insight into the perceived regulatory challenges and opportunities for basket trials in drug repurposing for rare diseases, a multi-stakeholder dialogue was organised in March 2025. The aim of this dialogue was to contribute to a better alignment of stakeholder perspectives to accelerate therapy development for rare diseases, by identifying and mapping challenges and opportunities in three regulatory scenarios for basket trials in drug repurposing for rare diseases.

**Results:**

In total, 28 experts (regulatory, preclinical, clinical, patient, industry, HTA, and other) from eight European countries participated in an interactive online meeting. Thematic analysis of the meeting content showed the most important challenges and opportunities in navigating the regulatory scenarios, covering themes such as preclinical evidence in iPSC models, design of basket trials, involvement of patient organisations, regulatory roles and responsibilities, pharmaceutical product development, HTA and reimbursement, and societal views on repurposed drugs.

**Conclusions:**

This multi-stakeholder dialogue highlights the continued need for 1) early regulatory guidance for academic innovators, 2) a (re)calibration of regulatory roles and responsibilities, and 3) early creation of collaborative spaces involving patient organisations, HTA bodies and marketing authorisation holders in the development process to optimize the potential of innovative methods. This multi-stakeholder alignment is essential for the compliant adoption of iPSC models and clinical basket trial designs as promising accelerators for drug repurposing in rare diseases.

**Clinical trial number:**

Not applicable.

**Supplementary Information:**

The online version contains supplementary material available at https://doi.org/10.1186/s13023-026-04551-8.

## Background

While more than 300 million people are affected by rare diseases world-wide, only 5% have approved treatment options [1–3]. This lack of treatment for patients with rare diseases calls for innovative approaches in drug development. Drug repurposing, i.e. the redevelopment of existing compounds for new indications, is regarded as a promising method to accelerate and reduce the costs of the drug development process in the context of rare diseases [4–7].

An additional way to accelerate therapy development is to combine drug repurposing with a focus on similarities in the molecular and clinical manifestations across multiple rare diseases [8]. Drawing on the potential of these similarities, an innovative clinical trial design can applied: basket trials. Basket trials can be used to test the safety and efficacy of a single compound across multiple diseases or disease subtypes [9, 10]. They are increasingly used for drug repurposing in oncology, including patients with different tumour types yet involving the same mutation or biomarker [11, 12].

A similar approach is currently pursued in the SIMPATHIC project, which aims to accelerate drug repurposing for rare neurological, neurometabolic and neuromuscular disorders [13]. SIMPATHIC proposes acceleration by (1) studying the effects of compounds in induced pluripotent stem cell (iPSC) models and (2) considering a basket trial design using the same protocol for one compound in several indications with similarities in the molecular and clinical manifestations.

However, the absence of a single route to navigate the regulatory process is often mentioned as a challenge in drug repurposing [5, 6, 14]. The majority of drug repurposing projects start in academia, where there often is a perceived lack of regulatory expertise and/or experience [15–17]. In recent years, more regulatory guidance has been developed for academic researchers working on drug development. For example, a regulatory roadmap towards patient access for medicines in rare diseases was designed for academia, showing considerations for marketing authorisation (MA) and for alternative routes [18]. Furthermore, the EU initiative Strengthening Training of Academia in Regulatory Science (STARS) contributed to regulatory support and education [19]. For drug repurposing initiatives, the International Rare Diseases Research Consortium (IRDiRC) developed a Drug Repurposing Guidebook, including “building blocks” for regulatory activities [20]. In 2025, an overview of the possible routes towards market approval for repurposed drugs was published [21]. Although these sources provide an overview of the regulatory procedures towards patient access, none of these address specific challenges and opportunities for combining basket trials with drug repurposing to accelerate therapy development. Furthermore, it is currently unclear whether these regulatory frameworks provide sufficient guidance for academics in practice.

The limited regulatory expertise and experience in academia is enhanced by a lack of collaboration and alignment between stakeholders that is often seen as a barrier to success in drug development and repurposing [15, 16, 22]. Multiple studies show that multi-stakeholder collaboration is essential in treatment development for rare diseases [2, 23, 24]. Furthermore, the guidebooks mentioned above recommend to commence a regulatory dialogue at an early stage, e.g. by obtaining scientific advice (SA) or protocol assistance [18, 19]. Previous work also points out that a multi-stakeholder (and multicountry) perspective is one of the requirements to facilitate “regulatory readiness” for innovative clinical trial designs [25]. Thus, it is deemed important for new drug repurposing efforts to facilitate a dialogue with regulatory experts early in the project.

In practice, however, there is a lack of consensus on how and when to initiate such a dialogue with regulators and the best way to ensure patient access to a repurposed drug. For example, the EMA procedure for obtaining orphan drug designation (ODD) for one disease entity does not align with obtaining protocol assistance for a clinical trial including multiple diseases and eventually marketing authorisation for multiple indications. It is also unclear how clinical evidence obtained from real world data in off-label use of a drug repurposing candidate can be used in marketing authorisation procedures. Furthermore, it is uncertain how the new EU pharmaceutical legislation will affect drug repurposing, for example by requiring marketing authorisation holders to adopt the new indication in case of a successful application as proposed in Article 48 [26, 27]. The above issues create uncertainty around the regulatory procedures and considerations for innovative drug repurposing projects such as SIMPATHIC. Therefore, the aim of this study is to identify and map challenges and opportunities in the EU regulatory scenarios for basket trials in drug repurposing in dialogue with multiple stakeholders. Ultimately, these findings will contribute to a better alignment of stakeholder perspectives on the regulatory compliant implementation of basket trials in drug repurposing, to accelerate therapy development for rare diseases.

## Methods

A multi-stakeholder dialogue session was organised to explore three EU regulatory scenarios for basket trials in drug repurposing for rare diseases, and to identify and map the challenges and opportunities in each of the scenarios with a variety of experts.

### Participant sampling and recruitment

Experts were invited based on their knowledge of, involvement in, or experience with drug repurposing projects and/or innovative clinical trial designs and through the network of SIMPATHIC. Also, authors of recent publications on regulatory roadmaps for drug repurposing and/or rare diseases were invited. All participants consented to recording and transcribing the meeting and using their input for analysis and anonymised reporting in a scientific publication.

### Preparation of the meeting

**Fig. 1:**
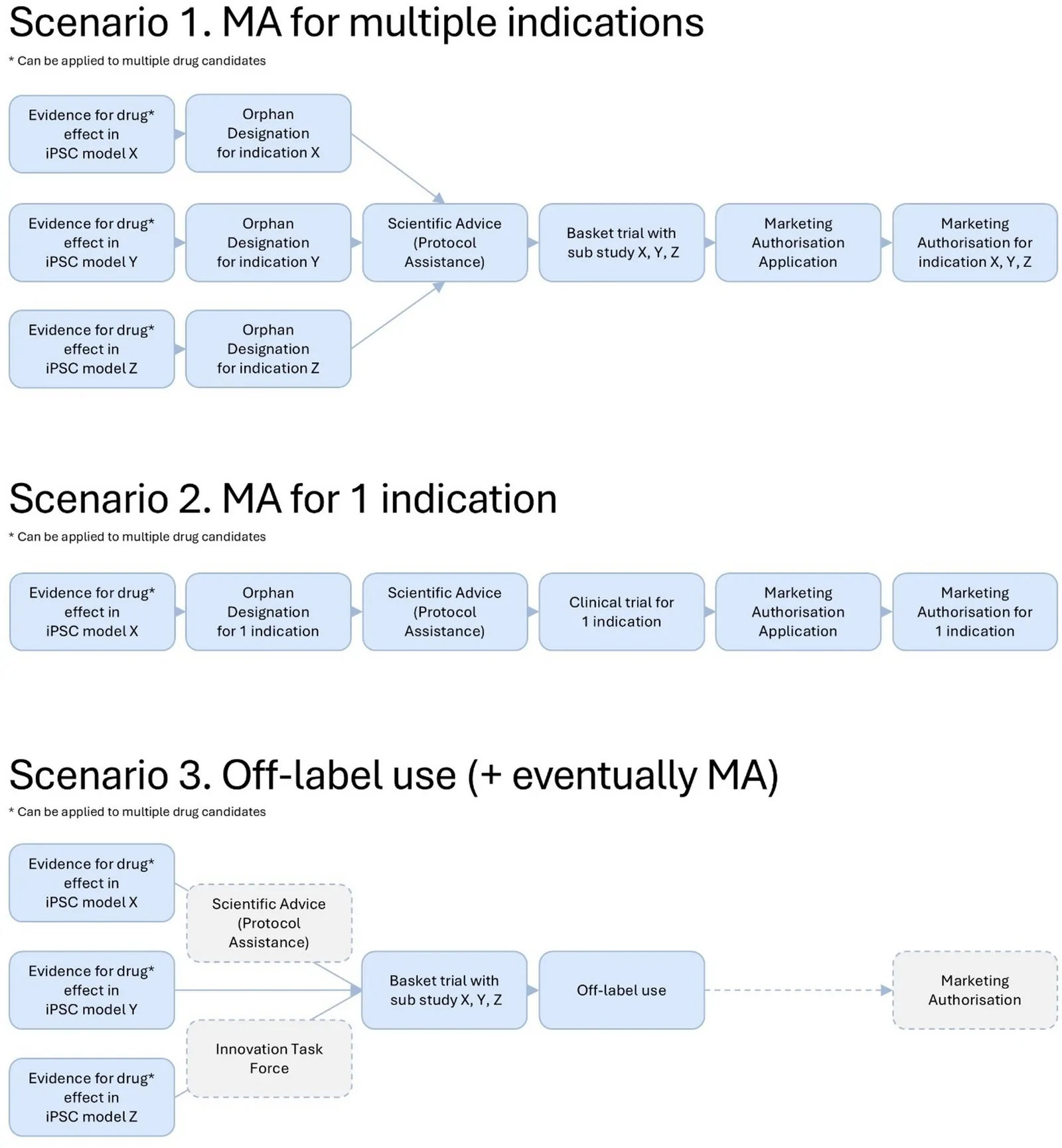
Overview of the three simplified European regulatory scenarios for basket trials in drug repurposing for rare diseases that were compiled in preparation for the multi-stakeholder dialogue session. Scenario (**1**) Preclinical evidence in iPSCs and clinical evidence in a basket trial results in marketing authorisation (MA) for multiple indications through a single regulatory process with one scientific advice and one marketing authorisation application. Orphan designation (ODD) is obtained for each indication separately. Scenario (**2**) The regulatory process is visualized for one indication resulting in marketing authorisation (MA). Scenario (**3**) Preclinical evidence in iPSCs and clinical evidence in a basket trial initially results in (temporary) off-label use with eventually marketing authorisation (MA) for the indications at a later stage. Here, obtaining scientific advice (SA) or interaction with an innovation task force (ITF) might occur in parallel or at a later stage. N.B. The regulatory scenarios should not be interpreted as mutually exclusive or as guiding roadmaps for basket trials in drug repurposing. Rather, the scenarios are intended to show the different routes that could be followed and to serve as a tool to make the associated challenges and opportunities explicit in the multi-stakeholder dialogue

In preparation for the multi-stakeholder dialogue session, we shared a document with the participants in which three simplified EU regulatory scenarios (Fig. 1) as well as associated considerations and questions were presented (Additional file 1). The scenarios were inspired by regulatory roadmaps for rare diseases and drug repurposing [18, 20, 21] and preparatory conversations with regulatory and clinical experts (*n* = 5) both internal and external to the SIMPATHIC consortium.

The scenarios reflect some of the relevant regulatory considerations for the use of basket trials in drug repurposing in the EU, along with questions and discussion points raised by involved stakeholders, specifically in the context of the SIMPATHIC project. These scenarios were shared with the rest of the participants to be reviewed three weeks prior to the dialogue session, where they served as a basis for discussion and for identifying further challenges and opportunities for basket trials in drug repurposing.

### Meeting procedure and analysis

The dialogue session took place on 10 March 2025 online via Microsoft Teams (2 h). The session started with a general and plenary introduction to the SIMPATHIC project and the aims and procedures of the meeting. Then, the different scenarios were explained to the participants, together with the identified points for discussion. After that, participants were divided into heterogenous subgroups in distinct Teams rooms with a facilitator and note-taker/observer (E.A.M.B., M.C.C., W.P.C.B., T.R.). In each subgroup, participants were asked to individually share their thoughts on digital post-its on a Microsoft Whiteboard. If possible, the post-its were placed in the scenarios where their considerations were seen as most relevant. Subsequently, the participants were asked to elaborate on the challenges and opportunities they identified, and other participants were encouraged to respond and share their input. In the concluding plenary part of the session, one person of each group summarized the most important challenges and opportunities identified in their subgroup with the other participants. To triangulate the findings from the subgroups, the other participants were asked to respond and indicate if important considerations were missing.

The notes and recording of the dialogue session were used to write a meeting report, which was validated by a member check. The input from session participants via Microsoft Whiteboards and the Teams chat, as well as a transcript of the meeting recording, were included in the analysis. The data were thematically analysed by E.A.M.B. following an inductive approach [28]. Emerging themes were continuously discussed in meetings with the co-facilitators of the dialogue session (M.C.C., W.P.C.B., T.R.) until consensus was reached. During the preparation of the manuscript, the description and hierarchy of the themes were further refined with all co-authors. Within each theme, the content was categorized as challenges or opportunities, and these were used to formulate four key considerations to accelerate drug repurposing for rare diseases, which were shared with participants for feedback and validation in a member check.

## Results

### Participants

**Fig. 2:**
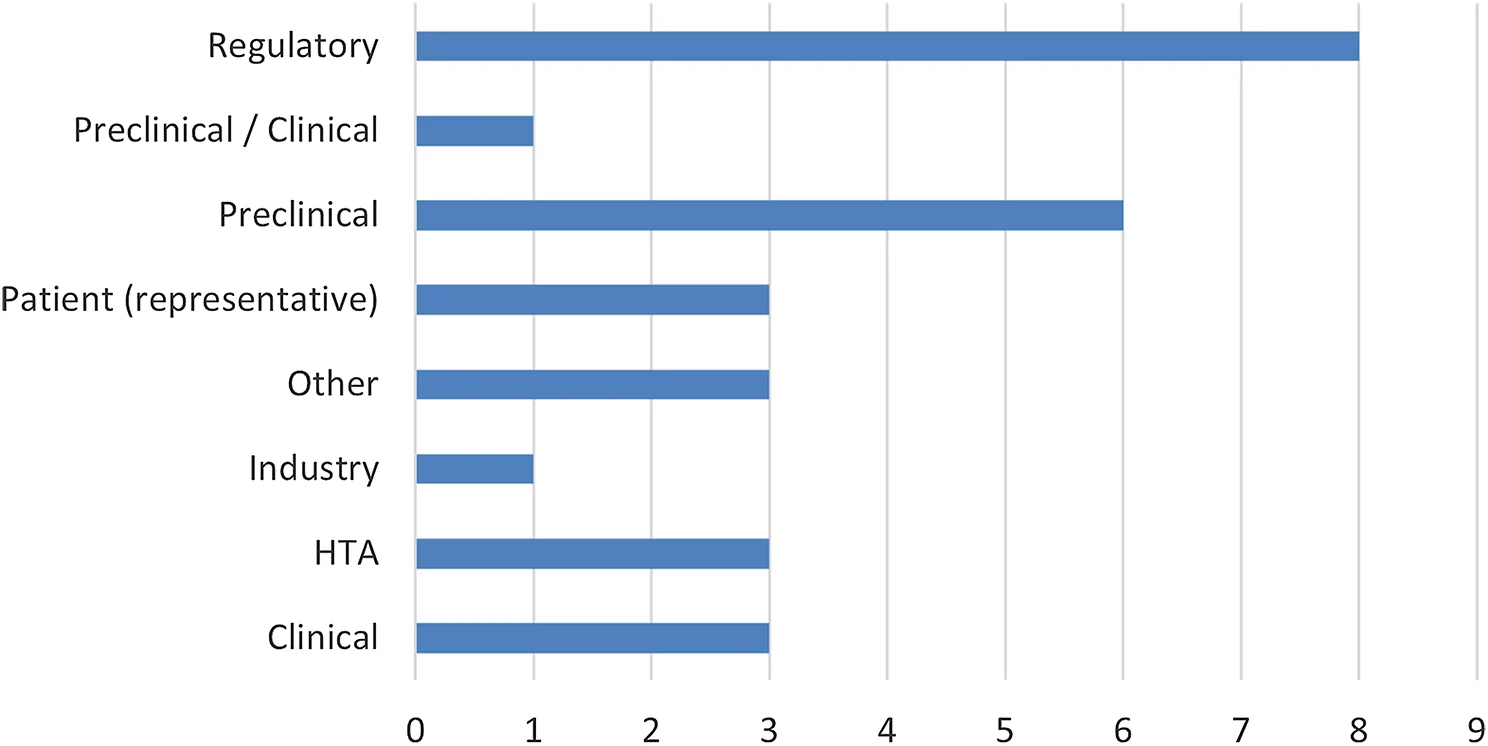
The number of participants per expertise* (regulatory, preclinical, clinical, patient representative, industry, HTA or other). *By ‘expertise’, we mean the area(s) in which the participants have the most knowledge and experience, which is not necessarily linked to their current professional or personal background. For example, participants with regulatory knowledge and experience are not necessarily regulators themselves

**Fig. 3:**
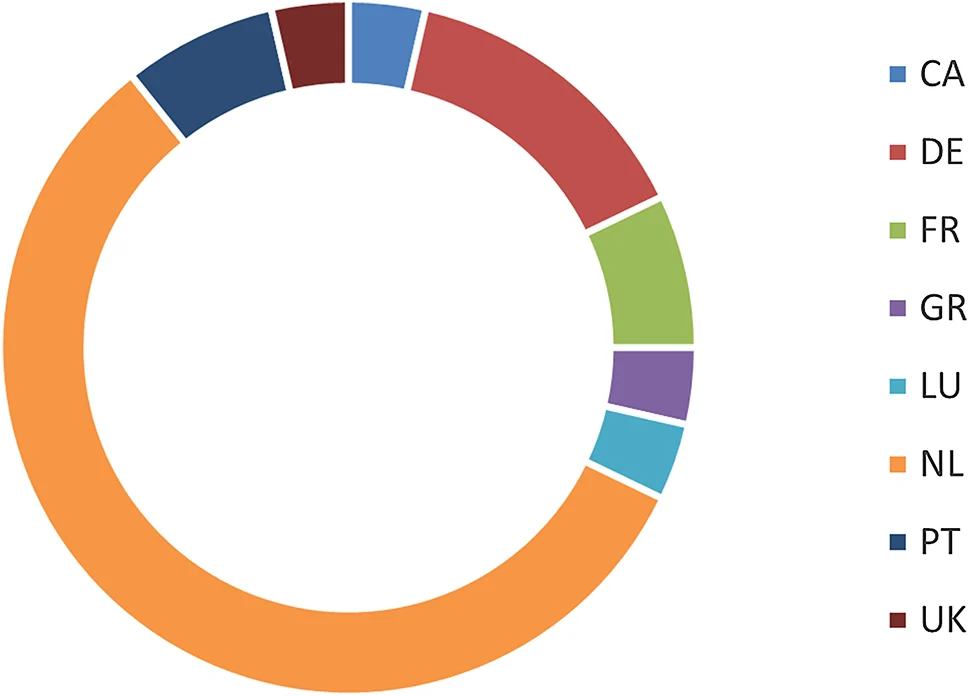
An overview of countries represented by participants in the meeting. CA: Canada [1], DE: Germany [4], FR: France [2], GR: Greece [1], LU: Luxembourg [1], NL: The Netherlands [16], PT: Portugal [2], UK: United Kingdom [1]

In total, 28 experts from eight countries participated in the regulatory meeting. In the figures below, an overview of the participants and their expertise is presented (Fig. 2), as well as their country of residence (Fig. 3).

### Challenges and opportunities

**Fig. 4:**
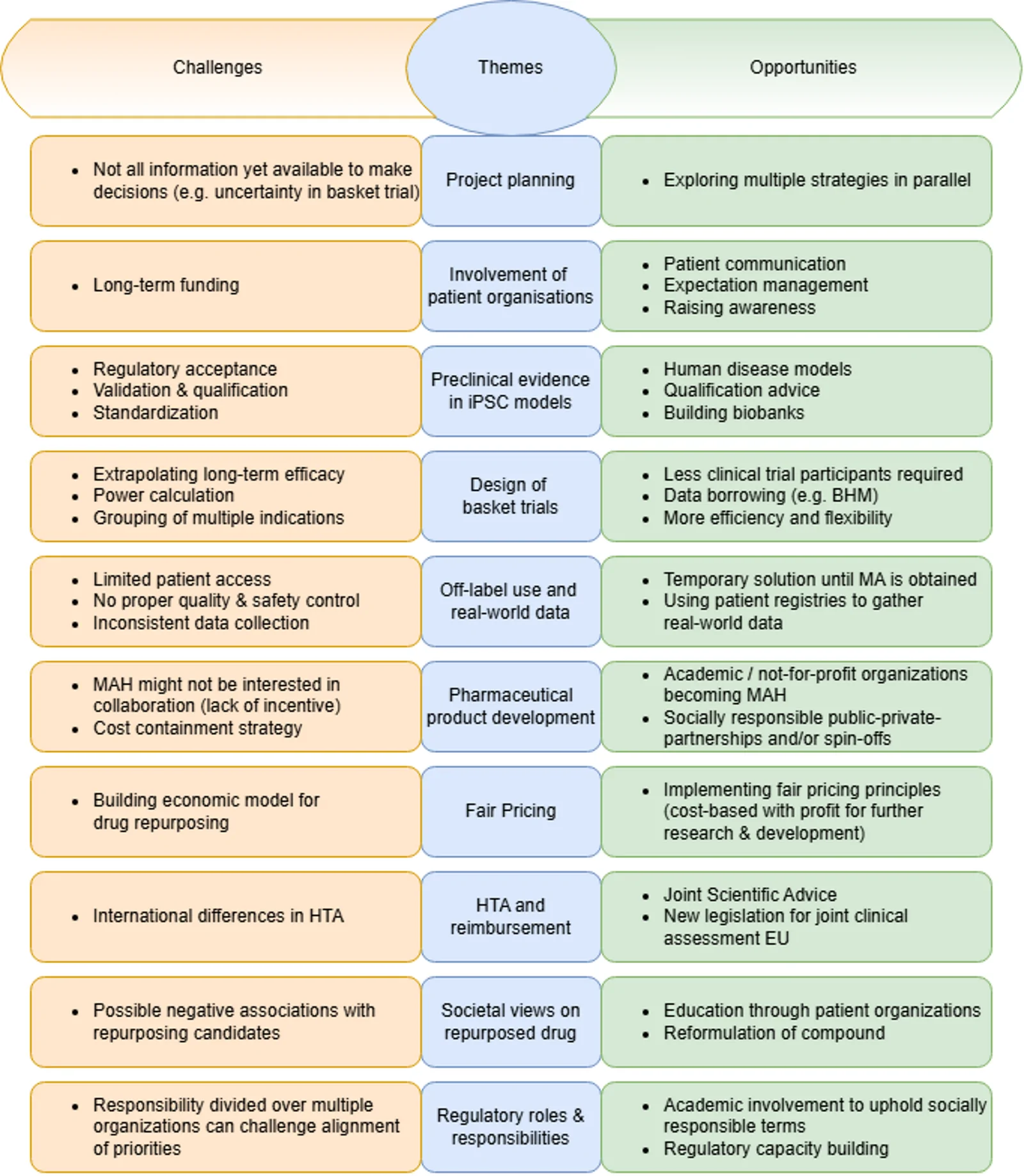
An overview of the challenges and opportunities for basket trials in drug repurposing that were raised and discussed by participants in the multi-stakeholder dialogue session. The challenges (in orange) and opportunities (in green) are described per theme (in blue). Abbreviations: BHM: Bayesian Hierarchical Modelling, MAH: Marketing Authorisation Holder, HTA: Health Technology Assessment

During the multi-stakeholder dialogue, several challenges and opportunities were proposed and discussed related to the different steps in the regulatory scenarios for basket trials in drug repurposing. The themes that were identified in the data analysis are summarized in Fig. 4 and are each described below.

### Project planning

An often overlooked, yet important challenge for drug repurposing projects is the project planning. Adding multiple sub-studies in one trial design may complicate the timelines for regulatory planning and evaluation. Session participants therefore remarked that it might be realistic to combine multiple regulatory strategies. For example, the SIMPATHIC project could start with scenario 2 (obtaining orphan drug designation, scientific advice, marketing authorisation for one indication) and add indications/sub-studies at a later stage. It was mentioned that a basket trial design should take into account such approaches (partially) in parallel, as inclusion and evaluation of sub-studies will likely not take place at the same time.

### Involvement of patient organisations

Continuous involvement of patient organisations was seen as a great opportunity in all steps of the drug repurposing process. They can contribute to defining patient reported outcome measures (PROMs), the organisation of clinical trials, patient recruitment, and working with patient registries. It was emphasized that patients can be strong advocates to raise awareness for drug repurposing projects and directly engage with important stakeholders. A challenge for the involvement of patient organisations is how to ensure long-term funding.

### Preclinical evidence in iPSC models

In SIMPATHIC, induced pluripotent stem cell (iPSC) models are developed for specific rare diseases to allow for functional studies as well as testing of multiple potential drugs in human cells. In general, using iPSC models for different purposes, mainly in the preclinical phase, was seen by the experts participating in the meeting as a great opportunity. They argued that iPSCs could serve as human models for rare diseases, e.g. by modelling a disease-specific genetic background, either with patient-derived or engineered iPSC models. Developing these models furthermore brings the opportunity to build biobanks and make disease-specific models available to further study rare diseases, for which animal models are often lacking. This can contribute to providing human-based evidence for drug repurposing.

From a regulatory perspective, it was recommended that iPSC models be qualified and validated for use in preclinical studies. It should be ensured that the model is fit for purpose, meaning that it is appropriate for the context of use. This also means that the model should be sufficiently complex, but not more complex than necessary. It was further recommended that the models be standardized to facilitate their application in basket trials with multiple diseases.^1^ Although there is growing interest from regulators in the use of iPSC models, some participants cautioned that seeking regulatory acceptance can be a lengthy and challenging process.

One important regulatory requirement is that there should be a clear rationale for using iPSC models (in vitro data) instead of animal models (in vivo data), for each indication separately. It was also explained by multiple participants to be aware of the different regulatory requirements for safety and efficacy testing, which may require different models. For example, for proof-of-concept studies an animal model may be preferred, but in vitro data may suffice if supported by a strong justification, e.g. when no suitable animal models are available and when the (patient-derived) iPSC model better recapitulates the human pathology on a cellular level. As pointed out by a regulatory expert, in practice, for dose finding studies an animal model is often requested to complete the preclinical dossier when applying for a new marketing authorisation, but it is not required for repurposing of an existing drug product when those data are available. Thus, for each step in the regulatory process, an accurate model must be used, supported by arguments as to why it fits the specific purpose.

The use of iPSC models as diagnostics was also discussed. The question was raised whether it would be essential to first demonstrate a similar molecular change in the iPSCs of individual patients before they become eligible for treatment with a specific drug (see also functional assays with organoids for cystic fibrosis [31] and cancer [32]). While the use of iPSC models as diagnostic tests could be seen as an opportunity to demonstrate their relevance, it was also acknowledged that it would require the development of patient-specific iPSC models for each potential clinical trial participant and could consequently delay the transition from the preclinical to the clinical stage. This would then compromise the acceleration envisaged in innovative drug repurposing projects such as SIMPATHIC.

### Design of basket trials

As proposed by the SIMPATHIC project, basket trial designs allow for the inclusion of multiple sub-studies representing different disorders or patient groups with similar symptoms and/or molecular mechanisms. Basket trials are increasingly common and considered as an interesting opportunity from both a regulatory and health technology assessment (HTA) perspective, as it allows for information borrowing to analyse the efficacy of a drug across multiple indications that have disease mechanisms in common. One participant pointed out that these analytical methods, such as Bayesian Hierarchical Modelling (BHM), have previously been used by HTA authorities for reimbursement applications for multiple indications in oncology [33]. Moreover, it potentially reduces the sample size required for inclusion in the clinical trial. Participants also noted that basket trial designs could make drug repurposing more efficient by providing the flexibility to add new sub-studies to a previously designed master protocol.

From a regulatory perspective, the justification of the trial design is particularly important. A number of regulatory challenges were identified for basket trial designs. First, it may be challenging to perform a power calculation with generic endpoints. Sensitivity in endpoints is essential to estimate the expected difference between drug and placebo. In addition, a crossover design makes extrapolating long-term efficacy more difficult. Another challenge that makes the extrapolation even more difficult is that the prognosis of patients with a specific mutation is often unknown. While there may be estimates from the overall disease population, these are not always considered relevant to translate into specific sub-indications. One participant suggested combining data from different disease registries as an opportunity to gain more insight into disease progression and to provide estimates for the development of the basket trial design.

Another challenge in the basket trial design is the difference between and within the different sub-studies. These differences are related to heterogeneity within the sub-indications, different patients and different age groups. For the trial design, this makes it challenging to define appropriate dose, formulation, and outcome measures. This may also prove challenging if there is a variation in efficacy between the different sub-studies. A regulatory expert therefore suggested not to rely too much on the basket trial alone but also allow for analysis in sub-studies separately.

In general, it was considered crucial to determine the most appropriate starting point for every regulatory procedure, which is the indication in the label (Sect.  4.1 of the SmPC: *Therapeutic indication(s) defining the target disease and the population to benefit from the medicine*). It is furthermore important to define this early in the project because this indication drives the clinical trial design, the inclusion of participants, the selection of endpoints, etc. The definition process can be facilitated by developing a Target Product Profile (TPP) with all relevant stakeholders providing input.

A related question is how to define a group of indications that is acceptable from a regulatory point of view. Participants agreed that to use such a definition for regulatory purposes, the underlying common factor should be well substantiated. The project could learn from tissue-agnostic indications that are used in oncology. For a disease-agnostic concept, a common disease mechanism, pathway or disease-causing biomarker needs to be clearly defined. The question was raised whether the common factor used to group multiple diseases could also be a clinical symptom. Even though it might be feasible to define a common symptom, e.g. “muscle weakness related mitochondrial disorders”, a known link between the symptom and underlying mechanism in each individual indication might still be required to clearly define common characteristics underlying the grouping of diseases. This demarcation problem was considered challenging for regulatory acceptance.

From a regulatory perspective, it was mentioned that the consecutive approval for multiple indications is more common, and this does not have to be a challenge as long as the efficacy of the drug is proven for each specific indication. It was also explained that the group definition can be dynamic, as there are examples of market authorisations becoming demarcated to sub-indications or splitting sub-groups based on new insights. This was e.g. the case for checkpoint inhibitors in oncology [34]. So even though it might be a useful opportunity to define a group of diseases as a consistent concept in the project and for regulatory purposes, this was not considered essential to ultimately obtain MA for multiple indications showing a positive risk-benefit.

### Off-label use and real-world data

Off-label use of a drug repurposing candidate was considered problematic by most participants for several reasons. First, with off-label use, there is generally a lack of systematic quality control through post-marketing studies and surveillance. Often lacking such systematic studies, off-label use is associated with limited and/or inconsistent safety and efficacy data which complicate a proper risk-benefit analysis. Second, without MA, equitable access of the drug to all people who need it is not guaranteed. In some countries, patients may not have access to the drug at all, and there might be costs and reimbursement issues. Furthermore, if the drug is not included in guidelines for treatment of the disease, the knowledge remains limited and may not reach the patient. Also, in some countries, physicians are not allowed to prescribe off-label. Third, off-label use was not considered a sustainable approach because it does not encourage companies to keep the drug on the market, which can be risky if, for example, the on-label indication does not generate sufficient profit. One participant emphasised that this could result in repurposing candidates being removed from the market at any time.

On the other hand, (temporary) off-label use of the drug could also be an opportunity if the data can be used to support a future MA. The practical and clinical challenge that needs to be overcome is that relatively costly clinical trials are needed for indications that are not prioritized by companies. Using existing patient registries or databases to collect these data was suggested as a potential opportunity. This could also be facilitated by the help of patient organisations. The question remains what is needed to use this data from a regulatory point of view. Moreover, such data will only be available for repurposing candidates that are currently approved and available, which limits off-label use for repurposing candidates that are not freely available, such as those drugs that are shelved by the original marketing authorisation holder (MAH).

### Pharmaceutical product development

An often-underestimated aspect in drug repurposing projects is early consideration of product characteristics, including the need for additional placebo development or reformulation, which may require revisiting the Investigational Medicinal Product Dossier (IMPD). It is important to not only consider who will produce the compound and placebo for the trial, but also who can market the compound as a product for the new indication(s) and how to avoid high pricing. This also includes a strategy for deciding on a potential (commercial) MAH to avoid complexities in production, for example when a new dose strength or formulation is required. For repurposing generic drugs, there may be multiple MAHs in play, which should be taken into account when choosing the right partnering strategy.

A known challenge in drug repurposing is the lack of economic incentives for commercial parties. The session participants recommended to work on business models from an early stage onwards, and consider how academia can benefit to do further investigations. If a commercial MAH is going to be involved, it is suggested to build a partnership model with a cost containment strategy. Socially responsible public-private-partnerships were mentioned as an opportunity to team up and provide a win-win situation for all parties involved. Another opportunity is the creation of a spin-off as a responsible for-profit company that commits to a fair pricing strategy. There could also be an opportunity for not-for-profit organisations to become involved as MAH and enhance regulatory capacity building. Therefore, it is essential to decide early what type of organisation will go for MA and how patient access will be realized.

Another difficulty shared by one participant was the process to obtain intellectual property rights (IPR) for a known substance in the EU, especially when it is not linked to one (new) indication. It was explained that obtaining IPR might be difficult for drug repurposing candidates targeting a specific mechanism of action (MoA) that can be present in multiple diseases. The lack of IPR may also compromise incentives for companies to collaborate.

Article 48 of the proposed new EU pharmaceutical legislation was discussed in one subgroup. The proposed article aims to lower barriers for academia and non-profits to contribute to extending treatment indications of existing authorised medicines [26]. The session participants doubted whether this would be a sustainable solution for drug repurposing. One participant argued that “surprising” existing MAHs with a required label extension was not the right way to go because it undermines collaboration with the MAH. Instead, it was recommended to contact the MAH early in the development stage to build a relationship based on mutual trust and align the development plan not only with the needs of patients, but also the viability of the business plan.

### Fair pricing

For involving a commercial MAH, the question was raised how to implement fair pricing principles where reimbursement is proportionate to the investment. Participants pointed out that a value-based pricing does not guarantee fair pricing principles. This is supported by literature explaining how value-based pricing can lead to prices that are too high to meet society’s standards [35, 36]. One possible solution mentioned by participants was to remain involved as an academic institution to maintain a cost-based pricing approach which also allows profits to be used for further research and development. Furthermore, participants advised to involve HTA experts early in the project and to perform cost-effectiveness analyses to assess the potential societal value of the repurposed drug.

### HTA and reimbursement

Another opportunity for applying the basket trial concept in drug repurposing is the new EU Health Technology Assessment (HTA) legislation starting in 2025 with the aim of providing patients with faster and wider access to medicines [37]. For example, these new rules require only one EU-level submission for joint clinical assessment. This is currently applied for oncology, but will also become available for rare diseases (in January 2028).

### Societal views on repurposed drug

In the dialogue session, it was also recommended to be sensitive to the social context of drug repurposing projects. For some drug candidates, there may be negative associations in society that the project needs to be aware of because it might impact uptake by patients. Session participants discussed ketamine as an illustration of a drug where the societal context is precarious [38]. The challenge is to address this framing so that the medical application of the drug is taken seriously by all stakeholders. For patient organisations, it could be an opportunity to provide education about the (new) use of the drug for patients and discuss information about the compound within the patient community. It was also suggested that negative connotations could be an extra argument when considering reformulation of the compound, so that you move away from the more common and potentially socially sensitive use of the drug.

### Regulatory roles & responsibilities

A general point of discussion related to multiple themes was the role of academic parties in the repurposing process when grouping multiple rare diseases according to a clinical basket trial study design. The question was raised whether, in addition to executing the clinical research, an academic organisation or hospital can and should ultimately also be the marketing authorisation holder. This may even be necessary if a company is not interested in filing for a new registered use of the compound. In theory, both private and public organisations can apply for MA, but they have to meet the same requirements in terms of good manufacturing practice (GMP), quality assurance, and post-approval data safety monitoring. This can be challenging for academic parties due to limited experience, expertise, infrastructure, time and resources, in particular when it involves reformulation of products. For some products, the dossier approved by EMA for the initial indication may need to be updated if the indication is expanded, because quality control that was sufficient historically may need to be improved to meet current standards. It can also be practically and legally challenging to scale up local production for a few patients to (sustainable) production for patients based on an EU licence.

In general, continuous involvement of academic parties and/or charities in drug development and active participation in regulatory processes was seen as an opportunity to keep a product on the market and accessible to patients, as done for example by AFM-Téléthon [39]. Another advantage of involving academic and charity organisations is that it opens the possibility to enforce socially responsible terms, such as reasonable pricing.

Specifically for drugs to be developed for multiple indications through basket trials, responsibilities are likely to be divided among multiple organisations. One participant pointed out that this can make it difficult to align priorities (e.g. to file for which indication first, or to define common clinical endpoints), as each organisation focuses on its own (disease-specific) expertise. Therefore, a clear description of regulatory tasks and responsibilities and a common goal in such projects is considered even more important. If an external party is approached as MAH, early connection and collaboration was recommended as an opportunity to agree on the development pathway, the clinical trial design, inclusion of indications and to engage with the Scientific Advice Working Party (SAWP) in accordance with the scope of the intended critical path of development.

## Discussion

The aim of this multi-stakeholder dialogue was to identify challenges and opportunities in three EU regulatory scenarios for basket trials in drug repurposing, specifically in the context of rare diseases. The participants discussed relevant themes in different stages of the drug repurposing process, ranging from considerations for obtaining preclinical evidence in iPSC models to important requirements for marketing authorisation, many of them similar to drug development in general. Some of these challenges have been previously reported in reviews on the barriers and facilitators in drug repurposing [22, 40, 41]. This multi-stakeholder meeting shows how these challenges are further complicated by uncertainty regarding novel methods such as iPSC models and basket trial designs. Here, we discuss the main findings related to accelerating drug repurposing and provide key considerations for basket trials in rare diseases derived from the meeting that could contribute to achieving optimal outcomes from innovative drug repurposing projects such as SIMPATHIC.

### A new role for academia and new partnerships to accelerate drug repurposing

The results show that the challenges in drug repurposing for rare diseases, such as a lack of interest from companies to invest in clinical trials, requires academic researchers to come up with innovative solutions. At the same time, there is a lack of expertise, time and resources for academia and other non-for-profit organisations to take the required regulatory steps to obtain marketing authorisation. Particularly when adopting new approaches, it is essential to leverage existing regulatory know-how, knowledge, and expertise in order to accelerate drug repurposing. For example, the multistakeholder dialogue session demonstrates the uncertainty around the regulatory requirements for the use of iPSC models as preclinical evidence. As explained by the participants, preclinical studies in iPSC models can support regulatory decision-making as long as the model is reliable for the defined purpose. However, this does not mean that formal validation of the iPSC model is necessary in order to use this data in the preclinical dossier. It means that a strong justification is required as to how the model answers a specific research question, such as a proof-of-concept or dose-finding study. The Center for Drug Evaluation and Research (CDER) of the FDA has published a draft guidance document (2026) for the use of New Approach Methodologies (NAMs) in drug development, defining four validation aspects: (1) context of use, (2) human biological relevance, (3) technical characterization, and (4) fit for purpose [42]. The EMA Guidelines on the principles of regulatory acceptance of 3Rs (replacement, reduction, refinement) testing approaches (2017) are currently being revised [43, 44]. Such guidelines are of great benefit to innovators working with iPSC models for drug repurposing in academia and beyond.

Although many innovative methods start in academia, regulatory and marketing activities are often not prioritized and incentivized by academic institutions. As a result, academic drug repurposing efforts often remain limited to off-label use (scenario 3), which may be a temporary solution, but is not considered ideal for achieving optimal access for patients [17, 45]. This may also lead to additional preclinical and clinical studies that need to be conducted when developers wish to apply for MA at a later stage, which is even more challenging for rare diseases due to a limited number of patients, some of whom already use the medication off-label.

To accelerate the possibility towards marketing authorisation, academic innovators are therefore encouraged to seek regulatory advice and determine a MAH to collaborate with as soon as possible, to prevent double work at a later stage. Although not discussed in detail during this dialogue session, it can be an important opportunity for academic and not-for-profit organisations to explore the possibilities for obtaining intellectual property rights (IPR) for the new indication(s) [5]. Collaboration between the public and private sector, for example in the form of public-private partnerships (PPPs), are recommended to share the risks and responsibilities of further drug development [40, 46–48]. Incentives for such partnerships can also be social impact bonds promoted by governmental organisations [47, 48] or novel types of societal investment structures such as steward ownership [49]. Funding opportunities may also come from not-for-profit organisations such as patient organisations, e.g. through crowdfunding or charity funds [40, 47, 48].

### Regulatory support and incentives to accelerate drug repurposing

To structurally overcome the challenges in drug repurposing for rare diseases, systemic change is required. Currently, successful drug repurposing projects often depend on contextual factors such as philanthropy or established connections with experts (“champions”). To increase the success of drug repurposing, regulatory awareness among academic researchers should be increased and their support should continue in a readily accessible way [18, 50, 51]. In this respect, the fee waiver for scientific advice for non-profit organisations introduced by the EMA in 2025 is a great opportunity for academic parties to seek early engagement. Article 164 of the new pharmaceutical legislation also aims to provide regulatory, procedural and administrative support to not-for-profit organizations and small and medium-sized enterprises (SMEs) [27]. The EMA has also recently completed a drug repurposing pilot to provide regulatory support for (academic) investigators. As part of this pilot, a virtual Repurposing Observatory Group (RepOG) has worked on developing a framework for not-for-profit organisations with essential regulatory components [52]. In a concluding report, the EMA recognized - in addition to challenges regarding preclinical and clinical evidence - the need for increased multi-stakeholder interaction in the drug repurposing ecosystem, such as engagement with MAHs [53]. Such multi-stakeholder engagement can be facilitated by platforms such as REMEDi4ALL [54] and REPO4EU [55].

Furthermore, new incentives should be created for SMEs and pharmaceutical companies to invest in innovative drug repurposing projects. An analysis by van den Berg, Hollak [56] points out how a lack of competition for orphan drugs contributes to high prices for rare diseases, suggesting the need for policy adjustments. Article 48 of the new pharmaceutical legislation can provide a route to obtain a label extension of the original MA containing the new indication for repurposed drugs, but questions remain around the implementation of such policies [26, 27]. For example, it is unclear who will be responsible for extending the marketing authorisation, and what will happen if there are multiple MAHs (which is often the case with generics). Furthermore, as discussed in the multi-stakeholder dialogue session, drug developers must be cautious not to undermine a trust-relationship with essential collaborators. A new dosage or formulation could also provide an incentive for developers to bring a repurposed drug on the market for a new indication. Other incentives for drug repurposing projects may come from public sector investment and the role of governments [21].

### Key considerations to accelerate drug repurposing for rare diseases

#### Continuously involve all relevant stakeholders

One of the key lessons from this meeting is to continuously involve all relevant stakeholders in the regulatory strategy. In line with observations of Petyko and colleagues [22], stakeholder perspectives on the challenges and opportunities in drug repurposing may differ, depending on their expertise. This indicates that alignment of stakeholder perspectives is essential when working on the implementation of innovative drug repurposing projects. To manage the complexity of drug repurposing for multiple indications according to a clinical basket trial concept, it is even more important to align the goals and interests of the different (public and private) parties involved and build fruitful collaborations early in the development process, with a common understanding of the critical development path. In our multi-stakeholder dialogue session, some key stakeholders and considerations were identified:

##### Patient organisations

Involve patient organisations and patient advocacy groups in all steps of the drug repurposing process, including the clinical trial design, patient recruitment, communication and expectation management. Especially for optimising the design of basket trials, the help of patient organisations is essential to define the common factor underlying the grouping of diseases and the relevant common endpoints, such as a core set of PROMs. During the clinical stage, patient organisations are needed to make efficient use of patient registries and real-world data. Furthermore, they can address societal views on repurposing candidates.

##### HTA bodies

Involve HTA agencies at an early stage, potentially in a joint Scientific Advice. Basket trial designs require innovative statistical methods for which early dialogue with HTA experts is an opportunity to determine the best strategy to demonstrate cost-effectiveness. To make the repurposed drug globally available, it is important to know what evidence is needed for reimbursement decisions, and adapt the basket trial design accordingly.

##### Marketing authorisation holder(s)

Determine early in development who will be the MAH for the provision of the trial medication and sustainable production of the repurposed drug. Think about building partnerships and setting conditions for market access. For repurposing generic drugs, a trust relationship with current MAH(s) may be essential to obtain the necessary data to file for a hybrid or full MAA. Early involvement of MAH(s) is even more crucial since basket trials - if successful - may lead to multiple indication extensions. Also, it is important to consider the responsibilities that comes with marketing a repurposed drug, such as risk management and post-marketing surveillance.

#### Provide a scientific rationale for preclinical models and the clinical trial design

For the regulatory dossiers, the scientific rationale for the preclinical and clinical design is most important. Provide a good argumentation why specific (iPSC) models are used, or why a specific trial design is chosen. Since basket trials are not (yet) commonly conducted for rare diseases and regulatory guidelines specific for basket trials enabling registration of multiple indications do not (yet) exist, scientific substantiation is key in order to align the research with regulatory requirements at an early stage. Also provide a rationale for the specific target condition(s) and the supportive scientific underpinning to maximise the chance to obtain (conditional) approval(s).

#### Be prepared and flexible to explore multiple strategies in parallel

A basket trial design requires a flexible and adaptive approach from all stakeholders involved. Therefore, it is recommended to consider different regulatory strategies, which can be explored in parallel. For example, it is possible and realistic to work towards MA for one indication and add other indications at a later stage.

#### Reverse planning can help to seize opportunities and deal with future challenges

Working with the final product in mind (using a TPP) is essential to determine which regulatory steps to take and how to deal proactively with the challenges and opportunities discussed [57]. It was also advised to determine at an early stage which indication(s) will be included in Sect. 4.1 of the SmPC. These considerations will determine outcomes of key decisions for the basket trial design, the IMPD, MAH involvement, etc. In line with this reasoning, it was also recommended to obtain Scientific Advice as soon as possible for the design of the basket trial.

Furthermore, for a basket trial design, alignment between the different sub-studies for marketing approval is essential. Since parallel inclusion is hard to achieve, this requires early planning and flexibility, both from the involved researchers and from the regulatory authorities (e.g. by facilitating conditional approvals).

### Strengths & limitations

This work addressed the regulatory knowledge gaps for basket trials in drug repurposing by providing an overview of the perceived challenges and opportunities in the specific context of SIMPATHIC, while at the same time providing a platform to facilitate multi-stakeholder collaboration and regulatory decision-making in the project. By focusing on both the content and process of the required steps to obtain marketing authorisation, we provide insight into practical considerations for academic researchers and other stakeholders involved in drug repurposing, with many aspects applicable to drug development in general. The diversity of experts participating in this multi-stakeholder dialogue allows issues to be viewed from various perspectives. The sample of participants in this multi-stakeholder dialogue session is not sufficient to provide a complete overview of all the challenges and opportunities for basket trials in drug repurposing for rare diseases. Additional regulatory expertise and guidance is required to navigate the regulatory landscape to successfully apply for marketing authorisation. Especially the perspectives from HTA experts and commercial/industry stakeholders deserve further attention. It is also important to note that the challenges and opportunities discussed in this paper represent personal perspectives, and may not be representative to the experience of others. Furthermore, other considerations might apply for regulatory scenarios outside the EU and for (shelved) drugs that have no active development program towards marketing authorisation [21].

## Conclusions

This multi-stakeholder dialogue highlights the need for continued early regulatory guidance, a (re)calibration of roles and responsibilities in navigating the regulatory processes, and early creation of collaborative spaces, including patient organisations, HTA bodies and marketing authorisation holders in innovative drug repurposing projects. We encourage not-for-profit organisations, academic researchers and other stakeholders that are generally underrepresented in the regulatory landscape to collaborate in drug repurposing and take an active role in capacity building. This multi-stakeholder collaboration and alignment is specifically important to address the additional regulatory complexities for innovative methods grouping multiple indications, such as preclinical evidence requirements in iPSC models, the design of basket trials, and regulatory roles and responsibilities when multiple public or private organisations are involved. Early consideration of regulatory requirements and adaptive planning are essential to optimally navigate the path towards marketing authorisation for innovative drug repurposing projects. Together with increased awareness and access to regulatory support as well as prioritizing and organizing incentives for public and private involvement in drug repurposing, this will contribute to accelerating treatment development for people living with a rare disease.

## Supplementary Information

Below is the link to the electronic supplementary material.

Supplementary Material 1

Supplementary Material 2

## Data Availability

Due to privacy considerations and in line with the Data Management Policy of the Amsterdam UMC, the original transcripts of the meeting are not publicly available. Requests and questions about the data can be addressed to the corresponding author.

## Footnotes

1 More information on the qualification procedure can be found through the New Approach Methodologies (NAMs) [29] and specific Qualification Advice [30] can also be obtained from the EMA.

## Abbreviations

BHM: Bayesian Hierarchical Modelling
EMA: European Medicines Agency
FDA: Food and Drug Administration
GAS: Goal Attainment Scaling
GMP: Good Manufacturing Practice
HTA: Health Technology Assessment
IMPD: Investigational Medicinal Product Dossier
IPR: Intellectual Property Rights
iPSC: Induced Pluripotent Stem Cell
ITF: Innovation Task Force
MA: Marketing Authorisation
MAA: Marketing Authorisation Application
MAH: Marketing Authorisation Holder
MoA: Mechanism of Action
NAMs: New Approach Methodologies
ODD: Orphan Drug Designation
PPP: Public-Private Partnership
PROMs: Patient Reported Outcome Measures
SA: Scientific Advice
SAWP: Scientific Advice Working Party
SMEs: Small and Medium-sized Enterprises
SmPC: Summary of Product Characteristics
TPP: Target Product Profile
