# Supplementary material for: Navigating regulatory scenarios for accelerating drug repurposing in rare diseases: a multi-stakeholder perspective on the challenges and opportunities
Source: Orphanet J Rare Dis. 2026 Aug 14;21:414. doi: 10.1186/s13023-026-04551-8 (PMC13756861; doi:10.1186/s13023-026-04551-8)
Supplement: Supplementary file 1 [file 13023_2026_4551_MOESM1_ESM.docx]

# Additional file 1. Mapping of questions in the EU regulatory scenarios for basket trials in drug repurposing
